# Two-Stage Classification of Future Knee Osteoarthritis Severity After 8 Years Using MRI: Data from the Osteoarthritis Initiative

**DOI:** 10.1007/s10439-024-03578-x

**Published:** 2024-07-09

**Authors:** Teemu A. T. Nurmirinta, Mikael J. Turunen, Rami K. Korhonen, Jussi Tohka, Mimmi K. Liukkonen, Mika E. Mononen

**Affiliations:** 1 Department of Technical Physics, University of Eastern Finland, POB 1627, FI-70211 Kuopio, Finland; 2 Science Service Center, Kuopio University Hospital, The Wellbeing Services County of North Savo, Kuopio, Finland; 3 AI Virtanen Institute for Molecular Sciences, University of Eastern Finland, Kuopio, Finland; 4 Diagnostic Imaging Centre, Kuopio University Hospital, The Wellbeing Services County of North Savo, Kuopio, Finland

**Keywords:** Knee osteoarthritis, Machine learning, MRI, Classification, Multiclass classification, Severity

## Abstract

Currently, there are no methods or tools available in clinical practice for classifying future knee osteoarthritis (KOA). In this study, we aimed to fill this gap by classifying future KOA into three severity grades: KL01 (healthy), KL2 (moderate), and KL34 (severe) based on the Kellgren-Lawrance scale. Due to the complex nature of multiclass classification, we used a two-stage method, which separates the classification task into two binary classifications (KL01 vs. KL234 in the first stage and KL2 vs. KL34 in the second stage). Our machine learning (ML) model used two Balanced Random Forest algorithms and was trained with gender, age, height, weight, and quantitative knee morphology obtained from magnetic resonance imaging. Our training dataset comprised longitudinal 8-year follow-up data of 1213 knees from the Osteoarthritis Initiative. Through extensive experimentation with various feature combinations, we identified KL baseline and weight as the most essential features, while gender surprisingly proved to be one of the least influential feature. Our best classification model generated a weighted F1 score of 79.0% and a balanced accuracy of 65.9%. The area under the receiver operating characteristic curve was 83.0% for healthy (KL01) versus moderate (KL2) or severe (KL34) KOA patients and 86.6% for moderate (KL2) versus severe (KL34) KOA patients. We found a statistically significant difference in performance between our two-stage classification model and the traditional single-stage classification model. These findings demonstrate the encouraging results of our two-stage classification model for multiclass KOA severity classification, suggesting its potential application in clinical settings in future.

**Supplementary Information:**

The online version contains supplementary material available at 10.1007/s10439-024-03578-x.

## Introduction

Management of the knee osteoarthritis (KOA) is extremely challenging, and it usually inevitably progresses to a stage where joint replacement surgery is the only option [1]. Thus, prior to the onset of irreversible symptoms and signs of the disease, preventative measures should be targeted at those who are at high risk for the onset of KOA. However, prevention is only possible if the progression of the disease can be predicted. Many research groups have recently developed various classification methods for future KOA based either on computational finite element models [2, 3], or machine learning (ML) algorithms [4–11]. Finite element (FE) analysis is a computational technique to solve physics-based problems using constitutive and governing equations. They have shown promise for classifying future KOA [12], but the most significant barrier to clinical implementation is the time it takes to make accurate subject-specific models with detailed three-dimensional geometries and workable FE meshes [3]. Compared to FE-based models, the use of deep learning algorithms together with medical images has seen a surge in popularity in recent years [9, 13]. Tiulpin et al., 2019 [7] classified the progression of KOA by using radiograph (X-ray) images and a combination of Deep Convolutional Neural Network and Gradient Boosting Machine. Their model yielded the area under receiver operating characteristic curve (AUC) of 81.0%, which can be considered excellent performance.

Although image-based approaches show potential (AUC > 0.8) in the test dataset, they often fall short in achieving a high enough level of accuracy (AUC < 0.7) for an independent datasets [14]. One possible reason is that image-based ML models are dependent on the software, preprocessing, imaging instrument, and settings utilized [15]. As a result, model generalizability degrades and performance may vary across clinics and over time as devices are upgraded. One possible solution could be using knee joint dimensions derived from medical imaging, as these measures are less affected by variations in software or imaging equipment. To the best of our knowledge, knee joint dimensions have never been considered an indicator of OA risk and are hence rarely included in KOA classifications. Consequently, there is a need for a new method that utilizes cost-effective and easily accessible features, while also attaining a high level of accuracy.

An imbalanced class distribution is a common issue in healthcare data [16], especially when classifying progression. Follow-up data are often collected from a sample of healthy individuals, of which only a minority develop the condition of interest within a specific time range. As a result, healthy participants are usually in the large majority, while diseased patients are in the small minority. A potential solution is under-sampling methods such as Balanced Random Forest (BRF) [17, 18], which is an ML model that under-samples the majority class in order to balance the class distribution. BRF is useful for binary classifications, but in multiclass settings with many majority and minority classes, BRF tends to focus on one minority class while ignoring the others [19]. Therefore, it is preferable to decompose multiclass problems into several binary problems. This is accomplished in the three-class case by using a two-stage classification that takes advantage of the ordinality of the classification task. It classifies the majority class first, and then the minority classes. The two-stage classification method allows for a more targeted classification of each class.

The capability to classify KOA into different severities is critical for development of personalized treatment plans and interventions. Multiclass classification is substantially more difficult than binary classification and is frequently disregarded. This is also evident in a vast number of studies [11, 20, 21] that focus only on binary classifications. The use of multiple classes allows for a more nuanced understanding of the disease and its progression. Furthermore, by avoiding a binary outcome, we avoid oversimplifying the complexity of KOA and acknowledge that the disease has different stages and manifestations. If KOA is only classified as a binary outcome (healthy versus diseased), a small change in one feature, such as weight, can lead to a classification shift from healthy to diseased. This oversimplification may compromise the model’s ability to generalize effectively, as it fails to capture the complex and nuanced features of OA, making it difficult to adapt the model to real-life situations. Therefore, in our study, we classified individuals into three categories based on Kellgren-Lawrence (KL) scale: healthy (*KL* = 0–1), moderate (*KL* = 2), and severe KOA (*KL* = 3–4, including knee joint replacement). Classifying KOA into multiple classes increases the model’s reliability, generalization, and correspondence to real-world scenarios.

The main goal of this study was to use a two-stage method to classify the severity of knee OA after an 8-year period in radiographically healthy adults. We trained different combinations of ML algorithms with various features that are easily obtained during clinical visits. The goal was to determine which of these features had the greatest classification performance. We hypothesize that the two-stage KOA classification performs better than the typical single KOA classification when we have an imbalanced class distribution and three ordinal classes. If easily measured features can be utilized to develop an accurate prognostic model, its scalability would provide a novel and straightforward way for classifying KOA in future, ultimately improving patient care. The model may be scaled to any tomographic imaging approach that reliably detects the bone, allowing us to identify knee joint dimensions. This would allow quantitative tool to evaluate personalized risk for the onset of KOA and show effects of different preventative measures such as weight loss.

## Methods

### Osteoarthritis Initiative Database

This study used data from the Osteoarthritis Initiative (OAI) database. Knee magnetic resonance imaging (MRI) (Sequence: SAG_3D_DESS, slice thickness = 0.7 mm, pixel size = 0.36 mm × 0.36 mm) scans were acquired in accordance with FDA guidelines, while knee radiographs were acquired in accordance with typical guidelines for annual and total radiation dosage for research subjects. Written consent was obtained from all subjects prior to each clinic visit. The OAI study was approved by the Institutional Review Board for the University of California, San Francisco, and its affiliates. The IRB approval was also obtained from all four clinical sites located at Brown University in Rhode Island, Ohio State University in Columbus, Ohio, the University of Maryland/Johns Hopkins University joint center in Baltimore, Maryland, and the University of Pittsburgh in Pennsylvania. Further details about the OAI data are accessible on the OAI website (https://nda.nih.gov/oai/). OAI data contained 4796 subjects, of which 683 (1213 knees) were eligible for our further analyses.

### Participant Selection

**Fig. 1:**
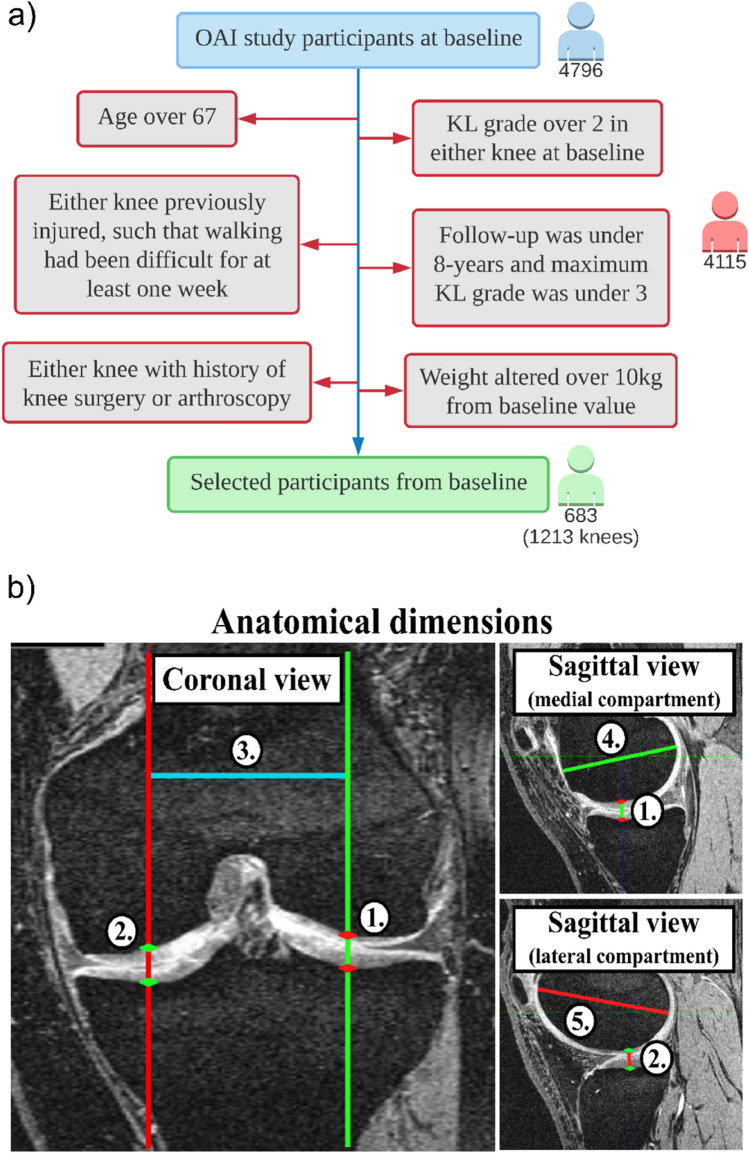
**a** Flowchart of participant exclusion criteria for machine learning model development and evaluation. **b** Determining anatomic knee joint dimensions (image features) from MRI images: 1 the medial tibiofemoral cartilage thickness (Medial JS), 2 the lateral tibiofemoral cartilage thickness (Lateral JS), 3 the distance between condyles (Condyle distance), 4 the maximum anterior-posterior length of the medial femoral condyle (Medial AP), and 5 the maximum anterior-posterior length of the lateral femoral condyle (Lateral AP)

In the current study, the focus was on the healthy working age population and therefore, the participants aged above 67 and participants with a KL grade of 3 or above in either knee at the beginning of the study were excluded. Furthermore, participants who had difficulty walking for at least one week due to a knee injury or had previously undergone knee surgery or arthroscopy were excluded from the study. This is because knee injuries are difficult to classify. Participants with no reported KL grade after 8-year follow-up were excluded, unless the KL grade was 3 or higher at any time during the follow-up period, which was justified by the progressive nature of the disease. Finally, participants whose weight changed more than 10 kg at any time during follow-up were excluded from the study. As weight is one of the relevant variables controlling the risk of onset and progression of KOA, the classifier was trained and tested in participants whose follow-up and baseline condition in terms of weight remained constant. The participant exclusion criteria are presented in Figure 1a.

### Knee Joint Measures from MRI

Knee joint dimensions (Fig. 1b) were calculated by using self-coded MATLAB graphical user interface. In this interface, medial and lateral joint spaces (JS) were determined using the sagittal slice that was considered to be at the center of tibiofemoral contact region. The distance between condyles (Condyle distance) was set to match with the sagittal distance between slices that were used for determining medial and lateral joint spaces. The maximum anterior-posterior (AP) length of the medial and lateral femoral condyle was again determined on the sagittal slice from where joint space was determined. Here, orientation of the anterior-posterior dimension was aligned respect with the ellipsoidal shape of the condyle so that it matched with the semi-major axis of the ellipse [12]. To prevent person-person variability, only one person conducted all knee dimension measurements in this study.

### Model Features

We trained our classification models based on 14 features (Table 1) that were assessed during the initial visit. The 14 features included age, gender, weight, height, walking pace, sit-to-stand performance, income, and smoking habits. These features have been found to impact KOA [22–27]. We also included knee joint dimensions from MRI images (Fig. 1b). Selected features were divided into five categories. The first category included basic subject characteristics (SC): gender, age, height, and weight. The second category included five knee joint dimensions (Medial JS, Lateral JS, Condyle distance, Medial AP, Lateral AP) from MRI images. The third category included physical performance features (walking and sit-to-stand pace). The fourth category included lifestyle features (annual income, and pack-years of smoking cigarettes). The fifth category included baseline KL grades based on radiographic findings. All included knees had KL2 or lower at the baseline. Target variables were maximum KL grade in eight-year evaluation (KL max). Targets were grouped based on the KL grades:

- KL01: healthy KL grades 0 and 1 (942 knees, 77.7% of total)
- KL2: moderate KL grade 2 (140 knees, 11.5% of total)
- KL34: severe KL grades 3, 4, and total knee replacement (131 knees, 10.8% of total)

**Table 1:** Each feature’s category, number, description, computed mean, and standard deviation (SD)

Category	#	Features: description	Mean ± SD
Subject characteristics features	1	Gender: male (*N* = 410 knees)/Female (*N* = 803 knees).	–
	2	Age: age at start of evaluation (45–67 years).	56.8 ± 6.1 years
	3	Height: average height (mm).	167.1 ± 9.0 mm
	4	Weight: average weight on scale (kg).	77.3 ± 15.7 kg
Knee measures from MRI (Image features)	5	Medial AP: maximum anterior-posterior length of the medial femoral condyle (mm).	58.4 ± 6.6 mm
	6	Lateral AP: maximum anterior-posterior length of the lateral femoral condyle (mm).	58.7 ± 6.2 mm
	7	Medial JS: medial side joint space (mm).	4.9 ± 0.9 mm
	8	Lateral JS: lateral side joint space (mm).	5.7 ± 1.0 mm
	9	Condyle distance: condyle distance (mm).	41.1 ± 4.2 mm
Physical performance features	10	Sit-to-stand pace: repeated chair sit-to-stand test: pace in (stands/sec).	0.54 ± 0.14 stands/sec
	11	Walking pace 20 m: 20-meter walk test: pace in (m/sec).	1.4 ± 0.20 m/sec
Lifestyle features	12	Smoking habits: pack-years of smoking cigarettes.	7.5 ± 14.7
	13	Income: annual income divided into five classes; less than $10 k, 2:$10 k to $25 k, 3:$25 k to $50 k, 4:$50 k to $100 k, and 5:$100 k or more.	3.9 ± 0.96
Baseline KL grade	14	KL grade: KL grade at baseline evaluation.	0.47 ± 0.85
Target	–	KL max: maximum KL grade in eight-year evaluation.	0.79 ± 1.2

The rationale behind merging KL grades was that greater differences between classes facilitate easier classifications by ML models. KL1 grade was merged with a healthy KL0 grade since it only shows doubtful joint space narrowing [28]. KL3 and KL4 grades have definite joint space narrowing [28], so they were also combined.

### Two-Stage Classification Model

**Fig. 2:**
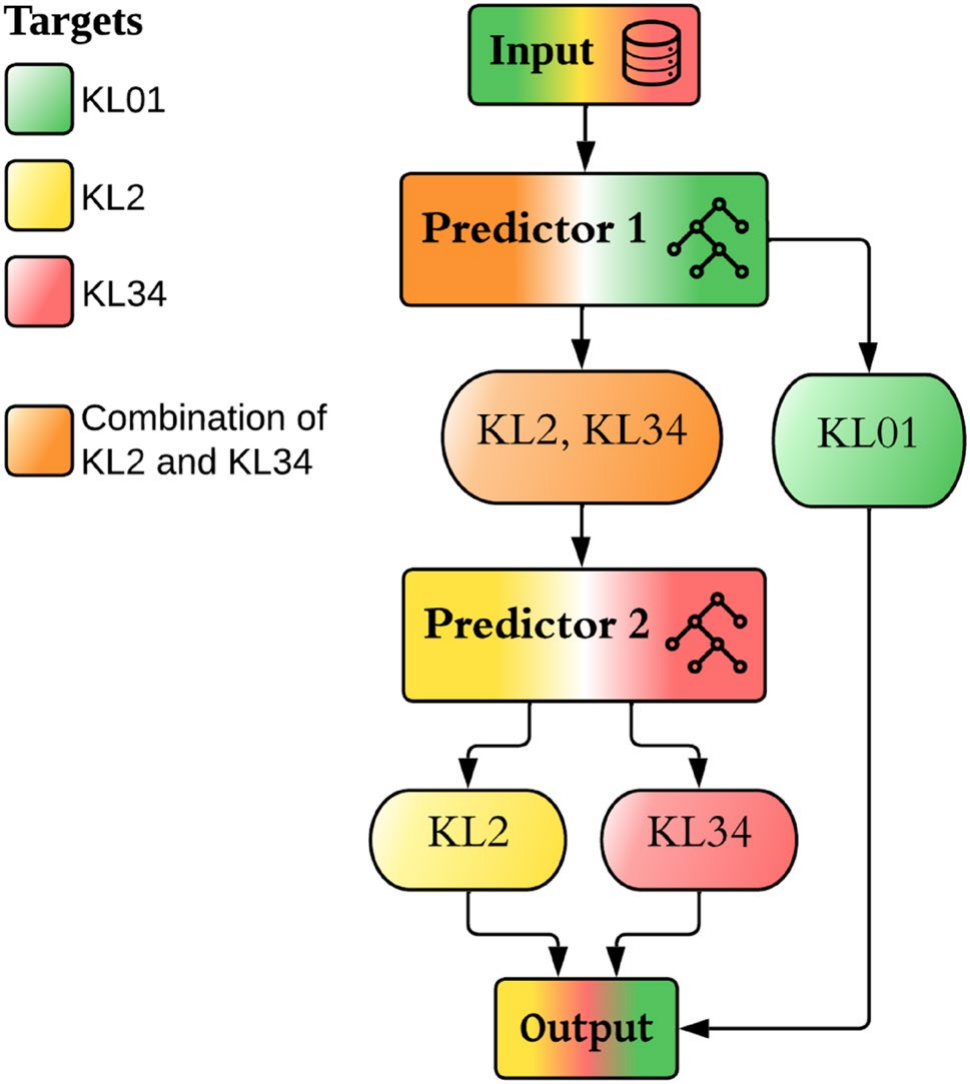
Flowchart of a two-stage classification model. KL01 and a combination of KL2 and KL34 are classified by the first-stage classification model. The classified KL01 values are output directly, while the KL2 and KL34 values are passed into the second-stage classification model, which classifies them into separate groups

We trained a two-stage classification model (Fig. 2). The first-stage classification model was trained using a dataset where minority class classes (KL2 and KL34) were merged into a single class. The reason for this was that the first-stage classification model can differentiate minority classes from the majority class (KL01). The training of the second-stage classification model involved the exclusion of participants whose target class was KL01. This was done in order to enhance the discriminative ability of the second-stage classification model in distinguishing between moderate (KL2) and severe (KL34) grades. Machine learning was done using Python (v. 3.9.7), Jupyter Notebook (v. 6.4.5), and the scikit-learn library (v. 1.2.2). We used 10-fold Stratified Cross-Validation [29] and two Balanced Random Forest [17] classification algorithms which were trained using 500 trees [18]. During hyperparameter tuning, we discovered that increasing the number of trees in the forest beyond 500 did not result in marked improvements. Furthermore, after experimenting with different maximum tree depths (3, 5, 10, 15, None), we observed that not limiting the depth worked best for our dataset. We chose conservative hyperparameter tuning to reduce the risk of overfitting. Feature importance was assessed using two different approaches: Scikit-learn permutation feature importance with 1000 permutations [29] and SHapley Additive exPlanations (SHAP) [30]. Permutation feature importance involves shuffling the values of each feature and measuring the impact on model performance, making it intuitive for interpretation. If features have high correlation (correlation matrix shown in Fig. S1), shuffling one feature may inadvertently affect the importance of another correlated feature. Thus, we have also established SHAP values, which do not possess the same limitation. SHAP is computationally intensive but can model intricate relationships between features and classifications, incorporating non-linearities and interactions for more detailed interpretations. Correlation analysis was performed in R (v. 4.3.1) using the corrplot and Hmisc libraries.

### Models

We studied five different models with different sets of features (Table 2). Two versions of every model were trained, one with and one without a baseline KL grade feature. Models with KL at the end of their names indicate that they employed the baseline KL grade. Model 1 was trained using SC (gender, age, height, and weight). Model 2 was trained using SC and knee joint dimensions from MRI (medial AP, lateral AP, medial JS, lateral JS, and condyle distance). Model 3 was trained using SC, physical performance (sit-to-stand test and walking pace), and lifestyle (smoking habits and income) features. Model 4 was trained by using all the features mentioned above. Model 5 was a reference model that used only one BRF algorithm rather than two. Model 5 was trained using the same features as in Model 1. The aim was to investigate if our two-stage models (Models 1-4) outperform the traditional single classification model (Model 5) approach when classifying imbalanced multiclass classifications.

**Table 2:** Each model’s name, features, and classification algorithms used for training

Models	Features (Table 1)	Without KL baseline	With KL baseline	Training data size	Classification algorithms
Model 1: subject characteristics (SC) approach	1–4, 14	Model 1	Model 1KL	1213	Two BRF
Model 2: SC and Image-driven approach	1–9, 14	Model 2	Model 2KL	1213	Two BRF
Model 3: SC, Physical performance, and lifestyle approach	1–4, 10-14	Model 3	Model 3KL	1054	Two BRF
Model 4: all features	1–14	Model 4	Model 4KL	1054	Two BRF
Model 5: single classification model	1–4, 14	Model 5	Model 5KL	1213	Single BRF

BRF refers to balanced random forest and SC to subject characteristics

### Performance Evaluation Methods and Statistical Analysis

**Fig. 3:**
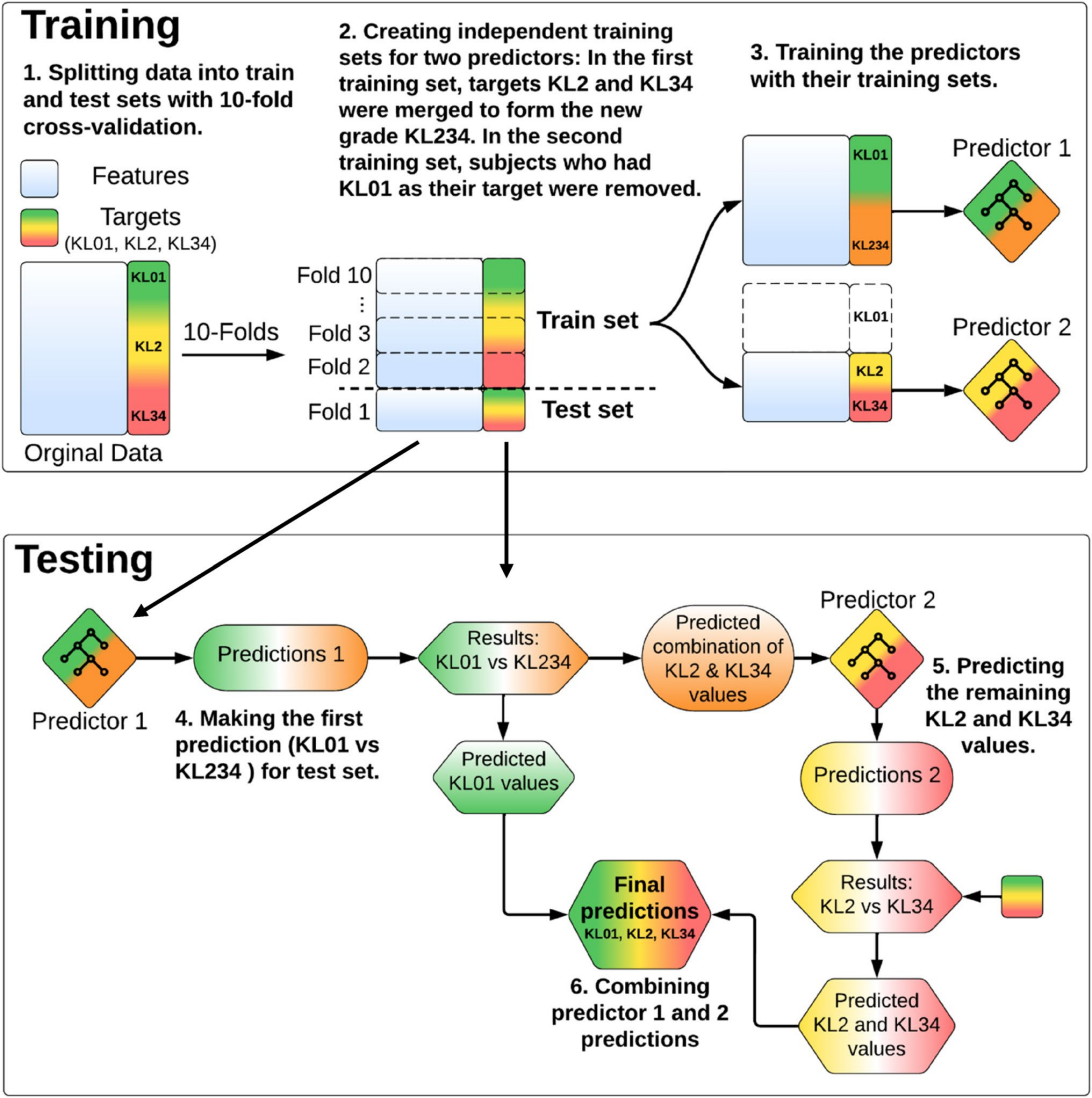
Flowchart of cross-validation training and testing for each fold. The training and testing processes are repeated 10 times, wherein one fold is alternated as the test set while the remaining folds are utilized as the training set

For evaluation, we employed Stratified Cross-Validation (CV) [31] with the constraint that the knees of the same individual were consistently grouped together in the same fold. This prevented a cases situation where data from a single person would be split across the training and testing sets (Fig. 3). To reduce the variance due to different train/test divisions, we repeated 10-fold CV 25 times and reported the confusion matrices and average performance measures across 25 repeats [10]. A confusion matrix is a table used for assessing the performance of a classification model by comparing its classifications to the true labels in the dataset. Variation was calculated by taking the square root mean variance of the fold-wise performance measure. In the evaluation process, we utilized scikit-learn [29], Balanced Accuracy (BA) [32], and Weighted F1 (WF1) [29, 33] metrics. BA takes imbalanced class distributions better into account and provides a more correct representation of the classification model performance than overall accuracy [16, 32]. The F1 score is a widely used metric for presenting classification results. In our evaluation, we have incorporated the weighted F1 score, which accounts for imbalances in class distribution. Also, AUC [34] was calculated for the first-stage (AUC 1) (KL01 vs. KL2 and KL34) and second-stage (AUC 2) (KL2 vs. KL34) classification models. AUC scores were evaluated in the binary case because multiclass AUC is challenging due to difficulty in deriving meaningful posterior probability scores. Also, we introduced AUC scores only because we compare them to existing studies that use binary classification models. These studies did not provide F1 or BA scores. To determine the statistical significance of the difference between models, we performed a paired two-sample *t*-test based on repeated k-fold cross-validation [35] with alpha level *p* = 0.01 defining significance.

## Results

**Fig. 4:**
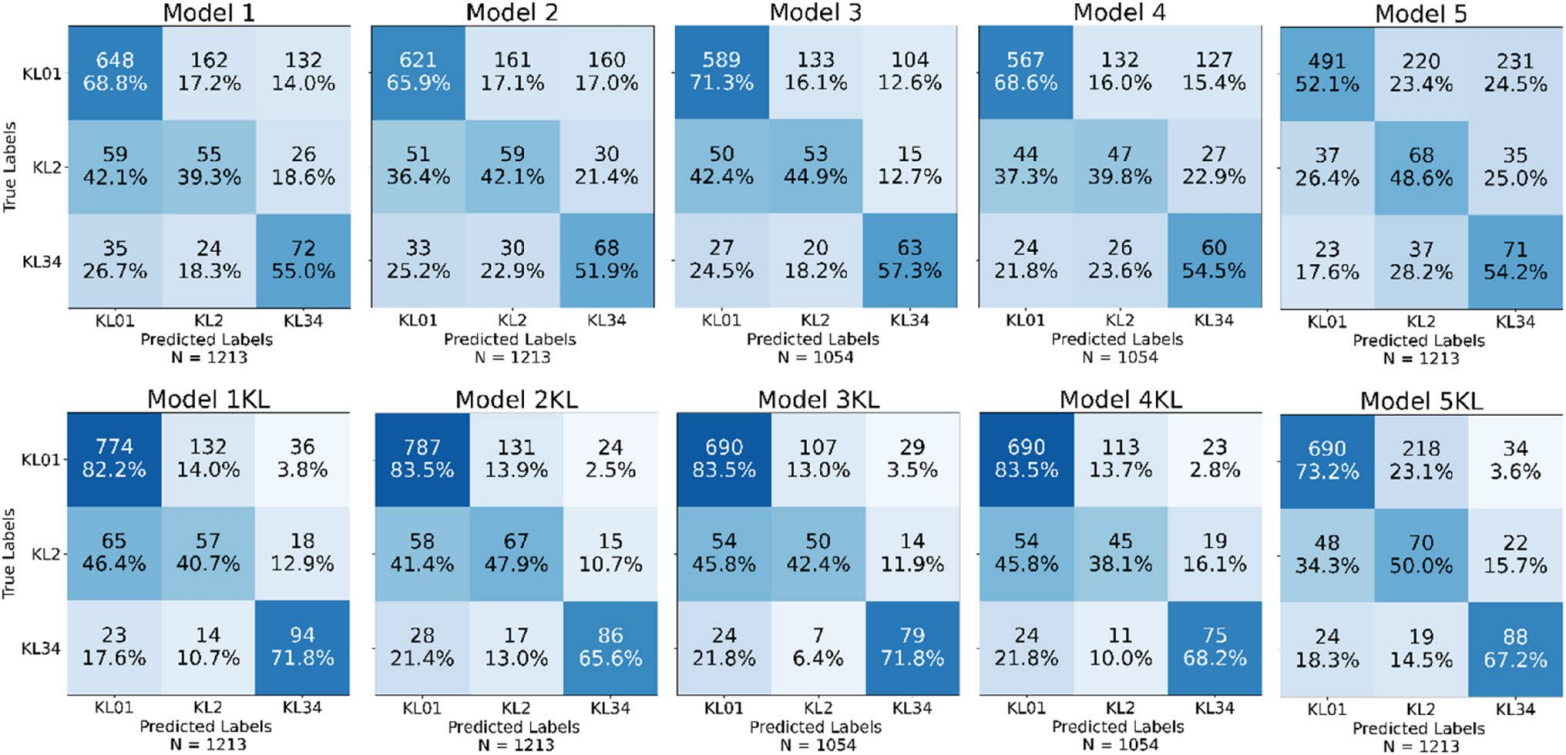
Models 1-5 and 1KL-5KL confusion matrices. Model 1 includes subject characteristics (SC) approach; Model 2 includes SC and Image-driven approach; Model 3 includes SC, physical performance, and lifestyle approach; Model 4 includes all features; and Model 5 includes Model 1 approach but instead of two-stage classification model (two classification algorithms), it uses single classification model (one algorithm)

When the baseline grade was not used, the BA and WF1 scores for Models 1–5 ranged from 51.6 to 57.8% and 57.7 to 70.0%, respectively. For Models 1–4, the AUC 1 ranged from 72.8 to 73.9% and the AUC 2 from 71.5 to 85.7%. When the baseline grade was included, the BA and WF1 scores for Models 1KL–5KL ranged from 63.3 to 65.9% and 73.4 to 79.0%, respectively. For Models 1KL–4KL, the AUC 1 ranged from 81.0 to 83.0% and the AUC 2 from 83.3 to 86.6%. Models that employed the baseline grade had on average, a WF1 of 11.4% and a BA of 10.4% greater than models that did not. Fig. 4 displays the confusion matrix for each model. Table 3 lists the scores for BA, WF1, AUC 1 and AUC 2.

**Table 3:** Balanced accuracy and weighted F1 scores for each model. Also, area under receiver operating characteristic curve (AUC) for the first-stage (AUC 1) and the second-stage (AUC 2) classification of each model. The error was computed by taking the square root of the mean variance of the performance measure across folds

	Model	Balanced accuracy	Weighted F1	AUC 1	AUC 2
Without KL baseline	Model 1	54.3 ± 5.6%	67.4 ± 3.6%	73.9 ± 3.7%	78.6 ± 6.9%
	Model 2	53.3 ± 3.1%	65.8 ± 3.0%	72.8 ± 5.9%	71.5 ± 1.4%
	Model 3	57.8 ± 3.8%	70.0 ± 0.9%	73.5 ± 2.5%	85.7 ± 5.6%
	Model 4	54.3 ± 2.9%	68.0 ± 1.6%	73.8 ± 3.6%	74.7 ± 5.4%
	Model 5	51.6 ± 3.4%	57.7 ± 2.0%	–	–
With KL baseline	Model 1KL	64.9 ± 5.0%	77.8 ± 2.9%	81.0 ± 2.9%	83.3 ± 6.6%
	Model 2KL	65.7 ± 4.1%	79.0 ± 2.8%	83.0 ± 4.7%	83.8 ± 6.0%
	Model 3KL	65.9 ± 3.7%	78.9 ± 1.6%	82.2 ± 2.1%	86.6 ± 8.4%
	Model 4KL	63.3 ± 4.5%	78.3 ± 0.9%	83.0 ± 2.8%	85.2 ± 8.4%
	Model 5KL	63.5 ± 6.5%	73.4 ± 3.7%	–	–

Two-stage classification models (Model 1 and 1KL) had significantly higher WF1 than the corresponding single classification models (Model 5 and 5KL). Model 5 and 5KL confusion matrices (Fig. 4) show that a single classification model appears to allocate higher weights to minority classes KL2 and KL34, resulting in a substantial number of KL01 values being categorized as KL2 and KL34. This decreases KL01 sensitivity while increasing the number of correct KL2 and KL34 classifications. If we use Random Forest (RF) (Fig. S2), which does not undersample the majority class, it tends to classify all classes into the majority class (KL01) when the baseline grade is not used. When baseline grade is introduced, RF appears to classify the majority of baseline KL01 patients into KL01 and most baseline KL2 patients into KL34, therefore gaining high KL01 and KL34 sensitivities while ignoring KL2. For Model 1, we also tested, RF [36], eXtreme Gradient Boosting (XGBoost) [37], and Easy Ensemble [38] classification algorithms (Fig. S3). RF and XGBoost performed quite similarly, with lower weights on higher KL grades resulting in a large number of KL2 and KL34 grades being classified as KL01. This increases KL01 classification accuracy while decreasing KL2 and KL34 classification accuracy. Easy Ensemble appears to have less rigid classification boundaries, resulting in more evenly distributed classifications. Many KL01 values are classified to be KL2 or KL34, whereas many KL34 values are projected to be KL1, lowering the overall classification. The performance of single BRF classification algorithms with different training sets is shown in supplementary (Fig. S4).

We tested our main hypothesis with a two-sample t-test based on repeated k-fold cross-validation to determine the statistical significance of the difference between our two-stage and single classification models. Our analysis’s findings show that there was no statistically significant difference between the models (1 vs. 5) and (1KL vs. 5KL) according to BA. The results showed that Model 1 had a BA of 54.3%, Model 5 had a BA of 51.6% (*p* = 0.11), and Model 1KL had a BA of 64.9%, and Model 5KL had a BA of 63.5% (*p* = 0.94). In contrast, when we examined the performance using WF1, we observed a notable discrepancy. Model 1 exhibited a significantly higher WF1 score of 67.4% compared to Model 5, which acquired a WF1 score of 57.7% (*p* < .00001). Similarly, Model 1KL showed a higher WF1 score of 77.8% compared to Model 5KL, which obtained a WF1 score of 73.4% (*p* < .00001). The performance of single BRF classification algorithms with different training sets are shown in supplementary (Fig. S4).

**Fig. 5:**
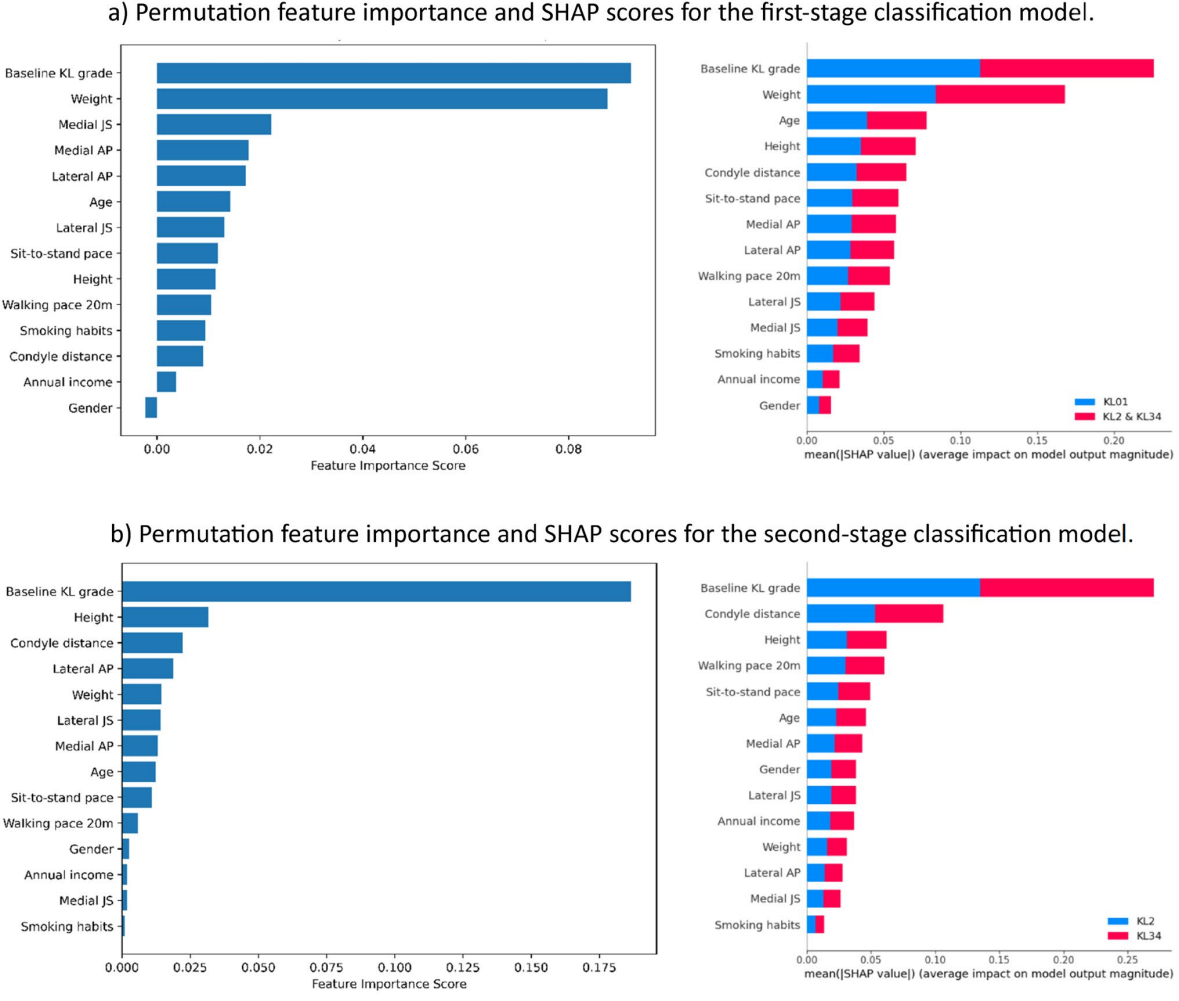
Permutation feature importance scores with 1000 permutations and shapley additive explanations (SHAP) scores for each class in **a** the first-stage and **b** the second-stage classifications of Model 4KL. SHAP illustrates how a feature impacts different classes. These impacts are shown in various colors and stacked to create a plot

We calculated permutation feature importance and SHAP scores for the Model 4KL first- and second-stage classification models to determine the important features. The model 4KL was chosen because it was trained using all the features presented in this study. Both methods revealed baseline KL grade and weight as the most important characteristics in the first-stage classification model (Fig. 5a). Gender, annual income, and smoking behaviors were the least important. In the second-stage classification model (Fig. 5b), the KL baseline grade remained the most relevant features, but weight was substantially less important than in the first stage. Height and condyle distance were important features in both approaches, although, in permutation, height was more essential than condyle distance, while in SHAP, the reverse was true. Medial JS and smoking habits had minimal importance in both techniques. Overall, both methods yielded similar results. Correlation matrix (Fig. S1) shows that all the knee dimensions are positively correlating with each other. As expected, KL baseline has the highest positive correlation with the maximum KL grade, while the weight has the second highest positive correlation. Sit-to-stand pace has the lowest negative correlation with maximum KL grades.

## Discussion

The main goal of the study was to utilize a two-stage ML method for classifying the severity of KOA after 8-year period among radiographically healthy adults. We trained multiple ML models with different combinations of features that are easily obtained during clinical visits. We aimed to identify which features contributed most significantly to classification performance. We hypothesize that our two-stage KOA classification approach would outperform the more traditional single KOA classification method, particularly when faced with an imbalanced class distribution and multiple classes. The overarching objective was to establish a more accurate prognostic model using easily measured features, thereby offering a novel and straightforward technique for classifying KOA and ultimately improving patient care. Our main findings were that the two-stage classification model seems to work better for imbalanced multiclass classification than typical single classification algorithms. We identified that KL baseline and weight were the most important features. Surprisingly, gender did not appear to have high importance. Knee joint dimensions did not significantly improve the classification performance, despite the fact that condyle distance had high feature importance when distinguishing between moderate and severe KOA cases.

We performed feature importance by using two different methods. Since moderate KOA (KL2) and weight are recognized as substantial risk factors for severe KOA [39], it was not surprising that the baseline grade and weight were the most important features in the first-stage classification model (Fig. 5a). Baseline grade possessed a higher contribution to the model performance in the second-stage classification (Fig. 5b), while weight importance decreased almost to zero. The most surprising result of feature importance was that gender had no effect on the performance of the classification models, even though it is generally accepted that female gender plays a major role in the increased risk of KOA [24, 40]. This could be explained by the fact that we had nearly twice as many females in the dataset as males. It is also possible that gender is related to other variables used as features in this study, such as joint geometry, which gender has strong negative correlation (Fig. S1), rather than being a risk factor itself. In the first-stage classification model, medial JS and AP had the highest importance, right after baseline KL grade and weight. In the second-stage classification model, condyle distance and lateral AP were the second and third most important features, respectively. The possibility of quantifying the effect of joint geometry in a healthy joint, including the joint space width, could help in the development of a KOA classification model.

Model 1 based on the SC performed well without the baseline grade feature and had a high KL34 grade sensitivity of 55.0% (Fig. 4). This is most likely due to weight being a high risk factor and therefore the algorithm values people with higher weight in the KL34 class. Model 1 trained with SC data outperformed Model 2 (SC and knee measurements), implying that knee joint dimensions may be noisy and reduce model performance. To investigate knee joint dimensions impact on classification, we trained an image-only models (supplementary Model 6 and 6KL) using solely the image features (Fig. S5). The image-only model (Model 6) had a BA of 40.3% and a WF1 of 55.7%. The AUC for the first-stage classifier (AUC 1) was 57.2% and for the second-stage classifier (AUC 2) was 64.6%. When the KL baseline feature was added (Model 6KL), BA and WF1 increased to 55.9% and 74.4%, respectively. The AUC 1 and 2 increased to 76.0% and 81.8%, respectively. Model 6 and 6KL performance metrics are marked in supplementary Table S1. Model 6KL performs reasonably well, but it is unclear whether additional features increase performance sufficiently to justify acquiring costly MRI scans. We already use radiographs to determine KL baseline grade, so we could attempt using radiographs to measure knee joint dimensions.

Model with SC, physical performance, and lifestyle features (Models 3 and 3KL) performed well in comparison with the others. High KL01 and KL34 classification accuracies indicate that physical performance tests aid in the classification of healthy patients. Individuals who performed well in these tests may be more active and hence have stronger muscles around their knee joint, which can aid in the prevention of KOA development. Model 3 additionally contained income and smoking habit features, which had a lower feature importance estimation than physical performance tests. Furthermore, Models 3 and 4 (with all features) had a reduced dataset since some participants (*N* = 159) lacked smoking habits and income features and were thus excluded. Different dataset sizes make comparing Models 3 and 4 to others more difficult.

In comparison with prior ML work [11], where the authors classified the KOA binary outcome in eight years using MRI imaging data, they obtained an AUC of 79.0%, whereas our equivalent Model 2KL (SC and image features) obtained an AUC 1 of 83.0%. In a recent multiclass classification study [10], they classified KOA progression into four different classes: one non-progressive and three progressive classes. Progressive classes were a progression of pain, structure, and a combination of pain and structure. They reported the results as a median WF1 score after performing cross-validation twenty-five times and taking the median WF1 score. This median WF1 score was 69.0%, while our corresponding WF1 value for Model 2KL was 79.0%. Our models perform well compared to the corresponding models in the literature, which further validates the reliability and applicability of our model.

All models were developed with and without the X-ray based baseline grade feature. KL baseline data helped to generate even better classification results in terms of AUC compared to classifiers without baseline grade input. As an AUC over 80% can be considered excellent classification accuracy [41], the developed method could already be applied as part of clinical decision making to assess the individual risk of developing KOA using a three-step classification (healthy, moderate, and severe). However, it should be noted that X-rays are often taken prior to MRI-based evaluations due to their cost-effectiveness [42]. When considering the automation of classification considering baseline grade status, there already exist numerous studies [13, 43–45] and applications that have automated KL evaluation from radiographs. Automated KL evaluation removes the need for a radiologist but not the need for an X-ray image. In future, we could focus on knee shape features that can be assessed from X-ray alone or different KOA classification systems such as WORMS [46] or MOAKS [47] that do not require X-ray.

There are potential limitations in our study. We did not validate our classification model using an external dataset, potentially introducing bias in the model’s performance. In future, the model should undergo testing using different external datasets. Our model’s primary limitation is that it was trained exclusively on a single data source. Although OAI data are gathered from various clinics in the USA, classifications may not be accurate for individuals of different ethnicities. Furthermore, implementing trained models in clinics can also be considered as limitation, since MRI is not a cost-effective method to examine KOA. In our current model, measuring knee dimensions is a laborious task, but measuring could be automated by example using convolutional neural networks in future. Overall, our two-stage classification model gave promising results for classifying multiclass KOA severity. More research is required before its promise can be fully realized. Our approach must be put into clinical practice and tested in a real-world study.

## Supplementary Information

Below is the link to the electronic supplementary material.

Supplementary file1 (PDF 1293 kb)

## Data Availability

The data created and analyzed during the current study are available from the corresponding author upon reasonable request.
