# Supplementary material for: Two-Stage Classification of Future Knee Osteoarthritis Severity After 8 Years Using MRI: Data from the Osteoarthritis Initiative
Source: Ann Biomed Eng. 2024 Jul 9;52(12):3172–83. doi: 10.1007/s10439-024-03578-x (PMC11560993; doi:10.1007/s10439-024-03578-x)
Supplement: Supplementary file 1 — Supplementary file1 (PDF 1293 kb) [file 10439_2024_3578_MOESM1_ESM.pdf]

Supplementary

Figure S1: The correlation matrix for all features listed in Table 1.

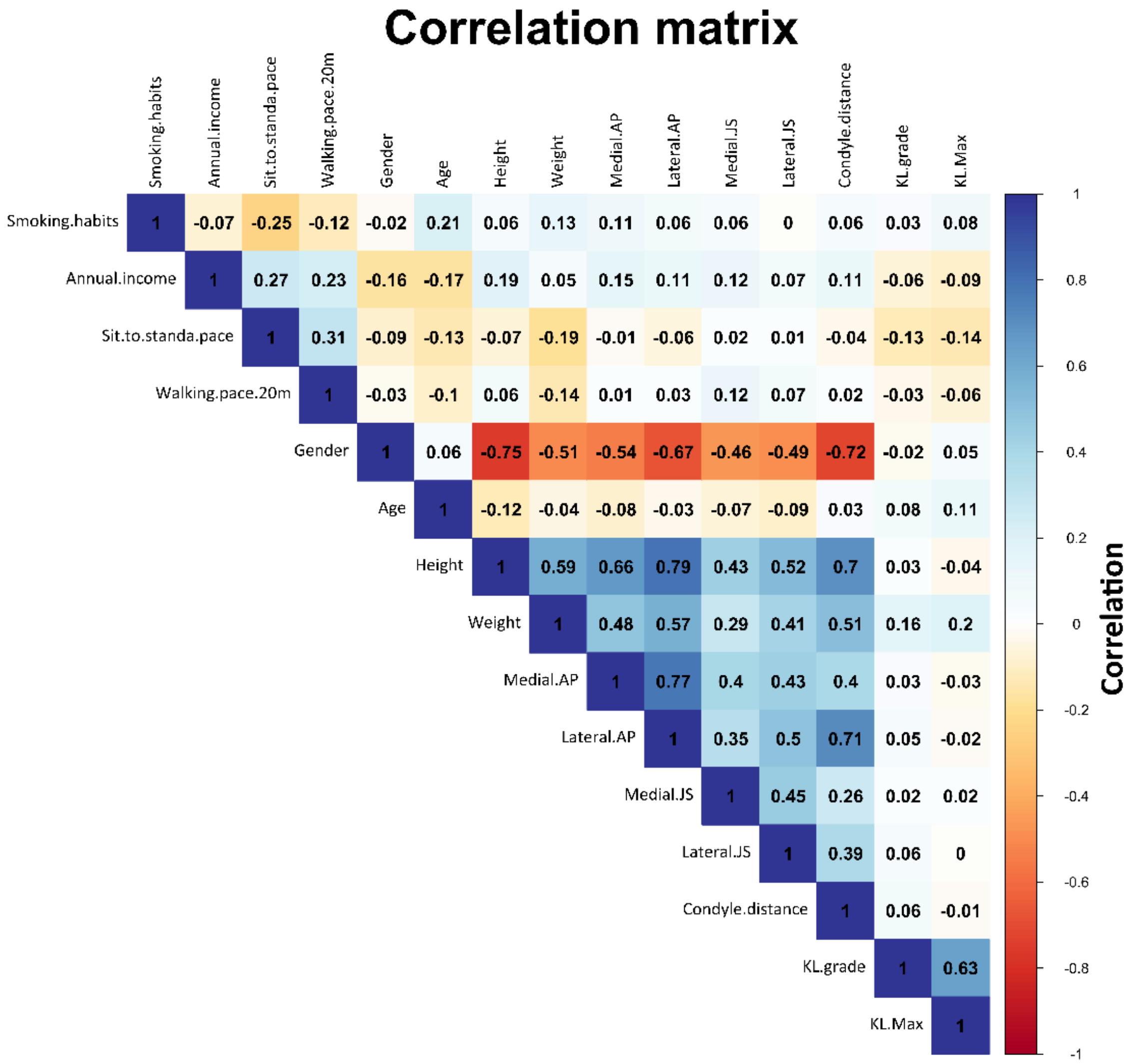

Figure S2: The confusion matrices of Model 5 and Model 5KL with singular random forest (RF) classification algorithm. We can observe that Model 5 with singular RF temp can classify everything to KL01 grade because KL01 is the majority class. Classifying everything to KL01 improves overall accuracy but completely fails to classify more interesting KL2 and KL34 grades. When KL baseline grades are introduced (Model 5KL), the model classifies that most patients with KL2 baseline grade will be classified to KL34 and everything else to KL01. Again, overall accuracy improves but model fails to classify KL2.

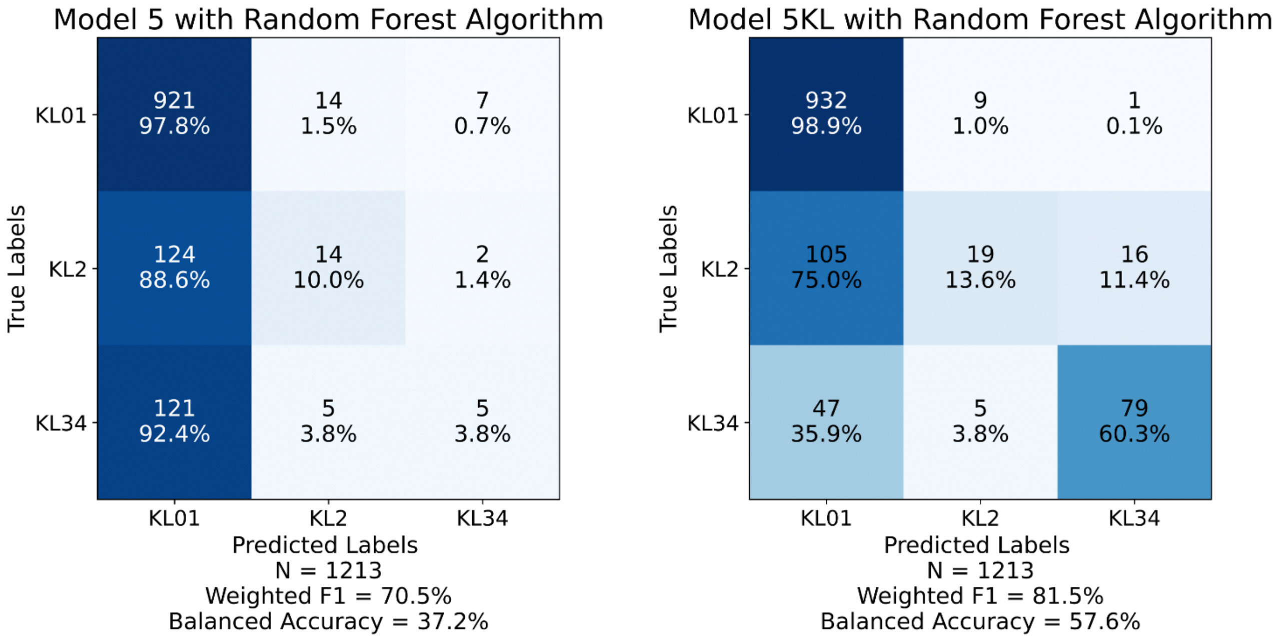

Figure S3: The confusion matrices of different classification algorithms with Model 1 and 1KL training data. a) Model 1 and b) Model 1KL with a two-stage random forest algorithm. c) Model 1 and d) Model 1KL with a two-stage extreme gradient boosting algorithm (XGBoost). e) Model 1 and f) Model 1KL with a two-stage easy ensemble learning algorithm. These algorithms lack the classification accuracy of KL2 grade, which was one of the reasons why we selected to use balanced random forest over these algorithms.

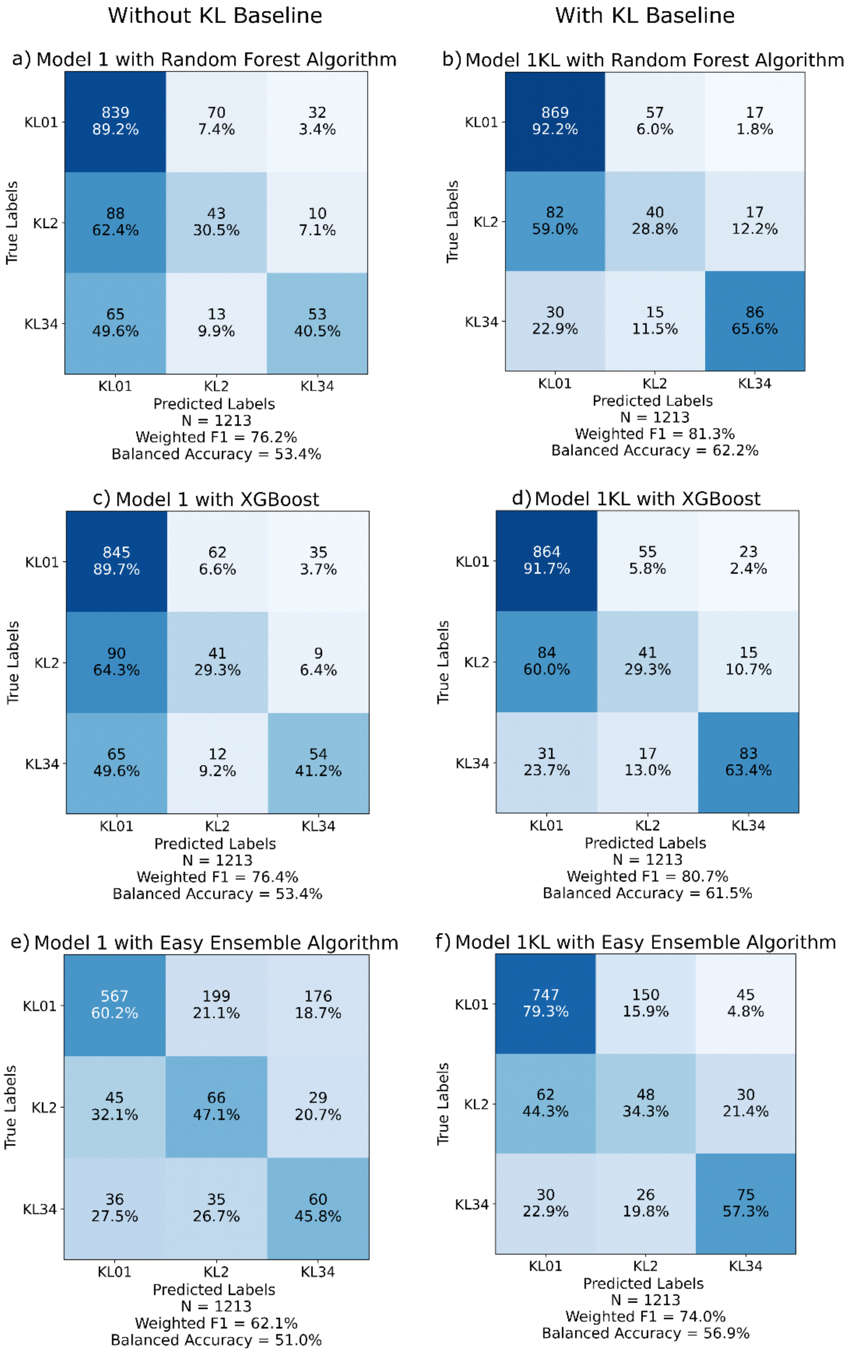

Figure S4: The confusion matrices of different models a) Model 2, b) Model 2KL, c) Model 3, d) Model 3KL, e) Model 4, and f) Model 4KL predictor sets when trained with single balanced random forest (BRF) classification algorithm. In our study, we compared two-stage and single BRF models with Model 1 and 1KL features. Therefore, here we provide single BRF models for other feature combinations (models). We can see that even with different features, single BRF model performance was worse than two-stage.

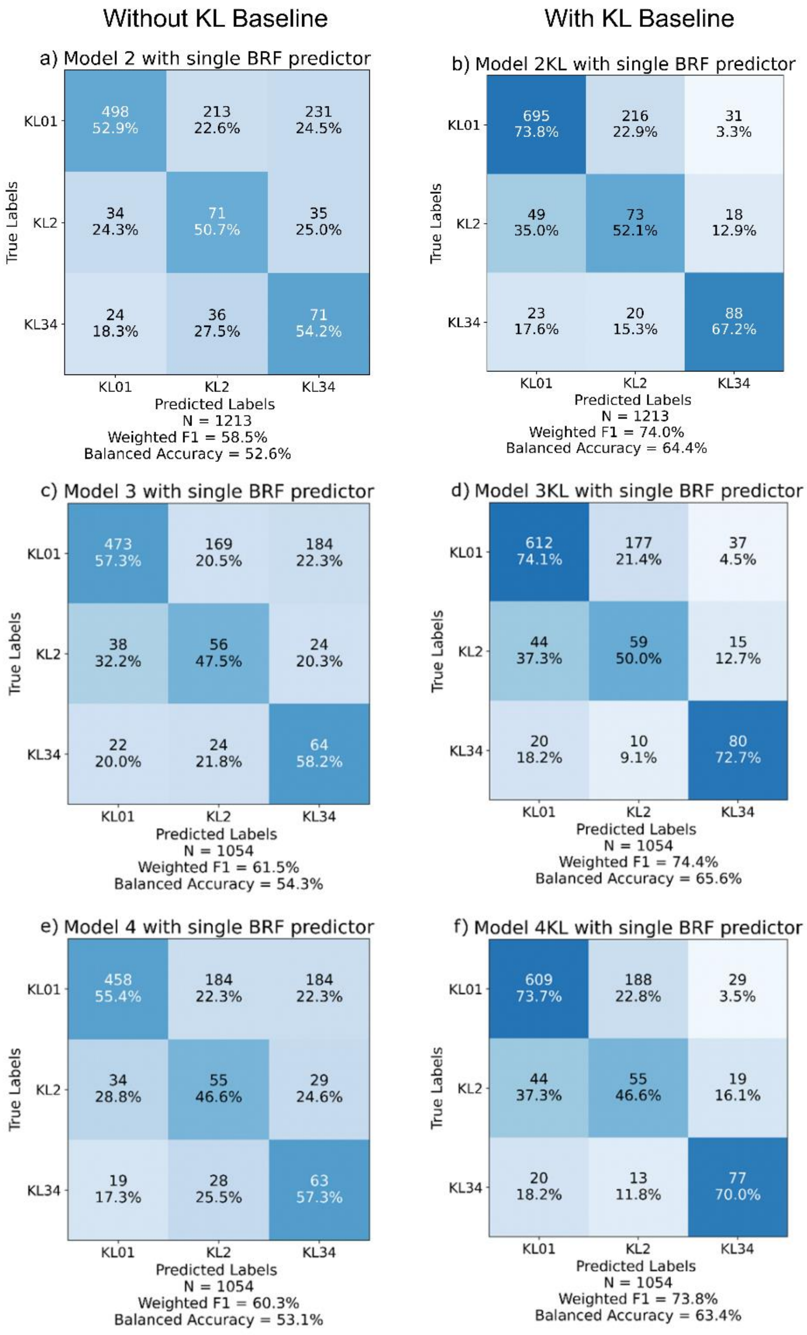

Figure S5: The confusion matrices of a) image-only model (Model 6) include features 5-8 from Table 1, and b) image-only model with KL baseline feature (Model 6KL) include features 5-8 and 14 from Table 1. Comparison of these results against to results reported in Figure 4 presented in the main text reveal the impact of knee joint dimensions on classification performance.

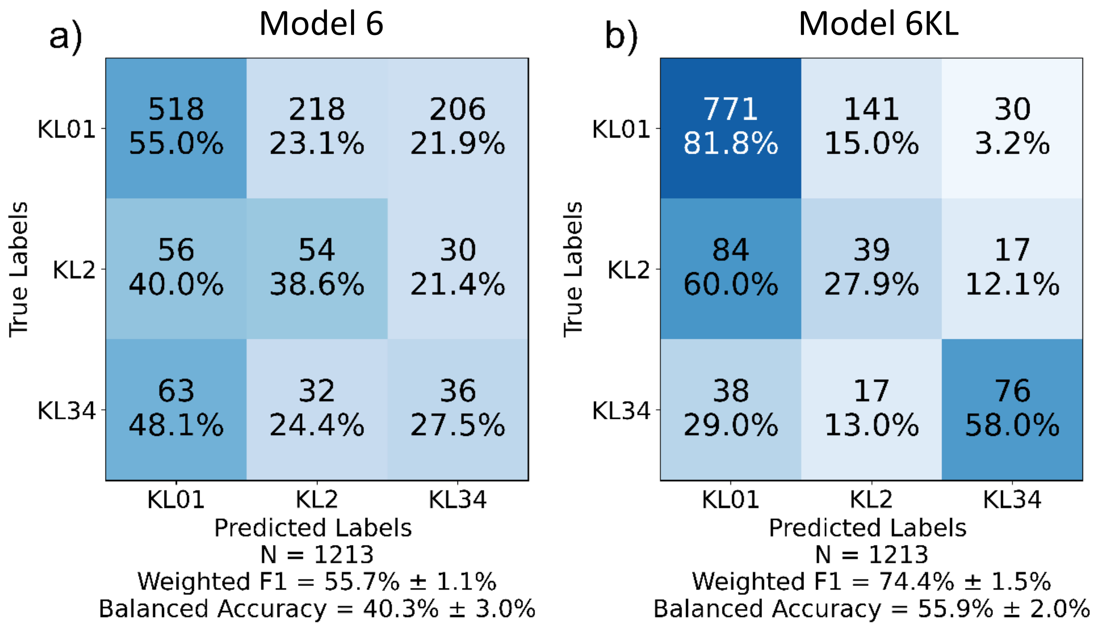

Table S1: Balanced Accuracy, Weighted F1, area under receiver operating characteristic curve (AUC) for the first-stage (AUC 1) and the second-stage (AUC 2) classification of image-only model (Model 6). The error was computed by taking the square root of the mean variance of the performance measure across folds.

Model	Balanced Accuracy	Weighted F1	AUC 1	AUC 2
**Model 6**	40.3 ± 3.0%	55.7 ± 1.1%	57.2 ± 8.0%	64.6 ± 9.9%
**Model 6KL**	55.9 ± 2.0%	74.4 ± 1.5%	76.0 ± 5.2%	81.8 ± 12.3%
